# Amelioration of sexual behavior and motor activity deficits in a castrated rodent model with a selective androgen receptor modulator SARM-2f

**DOI:** 10.1371/journal.pone.0189480

**Published:** 2017-12-07

**Authors:** Megumi Morimoto, Yuichiro Amano, Masahiro Oka, Ayako Harada, Hisashi Fujita, Yukiko Hikichi, Ryuichi Tozawa, Masuo Yamaoka, Takahito Hara

**Affiliations:** 1 Oncology Drug Discovery Unit, Pharmaceutical Research Division, Takeda Pharmaceutical Company Limited, Kanagawa, Japan; 2 CVM Drug Discovery Unit, Pharmaceutical Research Division, Takeda Pharmaceutical Company Limited, Kanagawa, Japan; 3 DMPK Unit, Pharmaceutical Research Division, Takeda Pharmaceutical Company Limited, Kanagawa, Japan; University of Louisville School of Medicine, UNITED STATES

## Abstract

Sarcopenia and cachexia present characteristic features of a decrease in skeletal muscle mass and strength, anorexia, and lack of motivation. Treatments for these diseases have not yet been established, although selective androgen receptor modulators (SARMs) are considered as therapeutic targets. We previously reported that a novel SARM compound, SARM-2f, exhibits anabolic effect on muscles, with less stimulatory effect on prostate weight compared with testosterone, in rat Hershberger assays and cancer cachexia models. In this study, we studied the mechanism of action for SARM-2f selectivity and also assessed whether the muscle increase by this compound might lead to improvement of muscle function and physical activity. First, we examined the tissue distribution of SARM-2f. Tissue concentration was 1.2-, 1.6-, and 1.9-fold as high as the plasma concentration in the levator ani muscle, brain, and prostate, respectively. This result showed that the tissue-selective pharmacological effect did not depend on SARM-2f concentration in the tissues. The ability of SARM-2f to influence androgen receptor (AR)-mediated transcriptional activation was examined by reporter assays using human normal prostate epithelial cells (PrEC) and skeletal muscle cells (SKMC). SARM-2f exerted higher activity against AR in SKMC than in PrEC. Mammalian two hybrid assays showed different co-factor recruitment patterns between SARM-2f and dihydrotestosterone. Next, we studied the effect of SARM-2f on motivation and physical functions such as sexual behavior and motor activities in castrated rat or mouse models. SARM-2f restored the sexual behavior that was lost by castration in male rats. SARM-2f also increased voluntary running distance and locomotor activities. These results suggest that tissue-specific AR regulation by SARM-2f, but not tissue distribution, might account for its tissue specific androgenic effect, and that the muscle mass increase by SARM-2f leads to improvement of physical function. Together, these findings suggest that SARM-2f might represent an effective treatment for sarcopenia and cachexia.

## Introduction

Androgens have wide variety of physiological actions including promotion of growth hormone release, stimulation of appetite, anabolic action, stimulus effect on the central nerve system, and regulation of energy homeostasis. Conversely, muscle loss, deterioration of physical activity, and lack of motivation occur as a consequence of several chronic diseases (cachexia) and normal aging (sarcopenia) [1–4]. As skeletal muscle provides the fundamental basis for human function, enabling locomotion and respiration, body wasting in the context of chronic disease is associated with reduced quality of life and impaired survival [5]. Accordingly, the development of cachexia or sarcopenia is generally associated with poor survival [6, 7].

Testosterone replacement therapy is effective for recovery of muscle mass and function in patients with sarcopenia or cancer cachexia [8, 9]. It has also been reported that testosterone exhibits stimulus effects on reproductive organs and physical activity in rodents [10, 11], which possibly reflects the clinical finding that testosterone improves late onset hypogonadism or physical activity in patients with sarcopenia and cachexia [12, 13]. However, testosterone replacement therapy has unavoidable side effects such as myocardial infarction, heart failure, stroke, depression, aggression, and aggravation of benign prostatic hyperplasia [14–17]. Therefore, the use of testosterone remains controversial. As an alternative, selective androgen receptor modulators (SARMs) have been intensively investigated for possible application in diseases with muscular weakness, although to date no SARM compounds have been launched as therapeutic agents for sarcopenia or cachexia [18]. We previously reported that a SARM compound, SARM-2f, selectively binds to androgen receptor (AR), stimulates AR transcriptional activity, and exerts an anabolic effect on skeletal muscles such as the levator ani muscle, soleus muscle, and gastrocnemius muscle in rat Hershberger assays and cancer cachexia models [19, 20]. In the present study, we attempted to elucidate the mechanism of action for the selectivity of SARM-2f by examining the tissue distribution of the compound, cell context-dependent AR transcriptional activity in reporter assays, and co-factor recruitment pattern in mammalian two hybrid assays. We also determined whether the muscle mass increase by SARM-2f demonstrated in our previous studies [19, 20] might lead to improvement of muscle function or physical activity in sexual behavior and motor activity tests. Our findings of tissue-specific AR modulation by SARM-2f and of SARM-2f-mediated physical function improvements suggest that SARM-2f might represent an effective treatment for sarcopenia and cachexia.

## Materials and methods

### Ethical statement

The animal protocol for this study was approved by the Animal Ethics Committee of Takeda Pharmaceutical Company Limited. In addition, all procedures were performed according to protocols approved by the Institutional Animal Care and Use Committee of the Pharmaceutical Research Division of Takeda Pharmaceutical Company Limited. The protocols were in accordance with the ethical standards laid down in the 1964 Declaration of Helsinki and its later amendments.

### Animals and compounds

Male, Sprague-Dawley (SD) International Genetic Standard-bred (IGS) rats were purchased from Charles River Laboratories Japan (Atsugi, Japan) at an age of 7 or 11 weeks. Female, Wistar Imamichi rats were purchased from SLC Japan (Shizuoka, Japan) at an age of 7 weeks. Male C57BL/6J mice at 7-weeks-of-age that were castrated or sham-operated at 6-weeks-of-age were purchased from CLEA Japan, Inc. (Tokyo, Japan). The animals were maintained under a 12-h light/dark cycle at a constant temperature of 23 ± 2°C with food (CE-2, CLEA Japan, Inc.) and water provided ad libitum.

SARM-2f (4-((2S,3S)-2-ethyl-3-hydroxy-5-oxopyrrolidin-1-yl)-2- (trifluoromethyl)benzonitrile) was synthesized at Takeda Pharmaceutical Company Limited. Testosterone propionate (TP) was purchased from Tokyo Chemical Industry (Tokyo, Japan). ALZET Osmotic pumps were purchased from DURECT Corporation (Cupertino, CA, USA), and Time Release pellets of dehydroepiandrosterone (DHEA) and TP were purchased from Innovative Research of America (Sarasota, FL, USA).

### Rat Hershberger assay

Male 12-week-old SD rats were castrated under isoflurane anesthesia. After eight weeks, they were divided into 12 groups (n = 4) based on body weight, lean body mass, and fat mass (day 0). A 1.5 mg/21-day release pellet of DHEA was implanted into each rat (day 0). SARM-2f was suspended in 0.5% methylcellulose (MC) solution and TP was dissolved in 20% benzylbenzoate (BB) in corn oil. The animals were then treated with the vehicle (0.5% MC, per oral (po.), once daily (QD), and 20% BB in corn oil, subcutaneously (sc.), thrice weekly), SARM-2f (0.02, 0.1, 0.5, 2, 10, or 50 mg/kg SARM-2f, po., QD, and 20% BB oil, sc., thrice weekly), or TP (0.1, 0.3, 1, or 3 mg/kg TP, sc., thrice weekly, and 0.5% MC, po., QD) for 4 weeks. Body weight was measured weekly and body composition was determined using nuclear magnetic resonance spectroscopy (EchoMRI-700, Echo Medical Systems, Houston, TX, USA) on day 0 and 27. On day 28, the skeletal muscles and sex accessory organs were removed and weighed. The rats were sacrificed under anesthesia after the Hershberger assay, and the organs were collected and weighed.

### Tissue distribution

**Fig 1:**
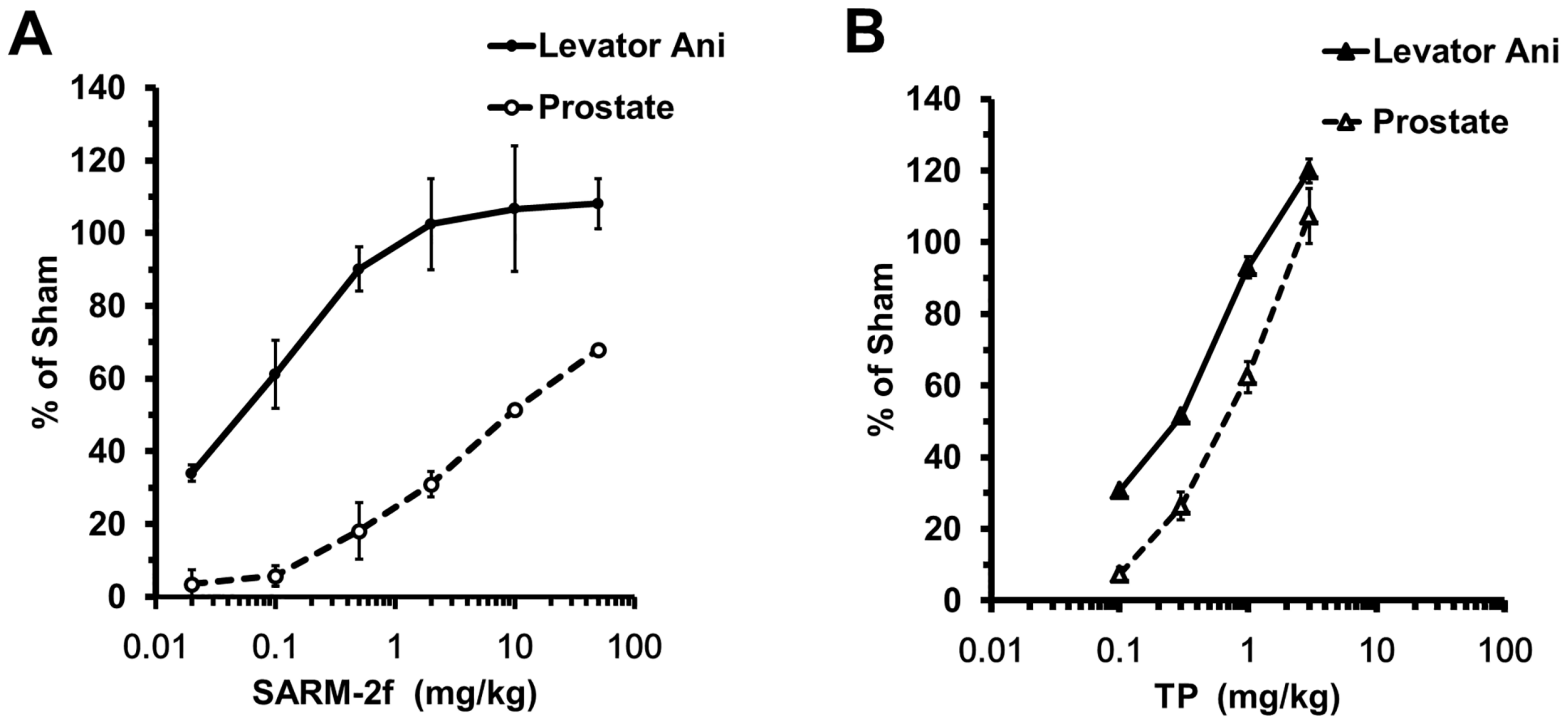
Tissue specificity of SARM-2f and testosterone propionate (TP) in castrated rats. Effect of SARM-2f (A) and TP (B) on the levator ani muscle weight and prostate weight. The y axis represents percent (%) of the sham group. The drug was administered to castrated male rats for three weeks. Data are shown as the means ±SD (n = 4–5).

SARM-2f at 0.75 mg/kg (suspended in 0.5% MC solution) was orally administered to eight-week-old male SD rats (n = 3). This dose is within the linear region of the dose-dependent curve of the weight of both the levator ani muscle and the prostate (Fig 1). Plasma or tissues were collected at 0.5, 4, and 24 h after the single dosing. The concentrations of SARM-2f were determined by liquid chromatography-tandem mass spectrometry (HPLC LC-20AD (Shimazu, Kyoto, Japan) and MS AB Sciex API5000 (Framingham, MA, USA)). The tissue-to-plasma concentration ratio (Kp) was calculated at each time point.

### Reporter assay in human prostate epithelial cells (PrEC) and skeletal muscle cells (SkMC)

Human PrEC and human SkMC were purchased from Lonza (Walkersville, MD, USA). PrEC and SkMC were cultured in special medium, PrEGMTM BulletKit (Lonza) and SkGMTM BulletKit (Lonza), respectively. Both cell lines were maintained at 37°C in 5% CO_2_ atmosphere. Dextran charcoal treated-fetal bovine serum was prepared as described previously [21]. PrEC and SkMC were cultured in medium without bovine pituitary extract and epidermal growth factor (steroid-reduced medium) on 6-well poly-L-lysine-coated plates at a density of 5.5 × 10^5^ cells for 2 days. Transfection mixtures containing AR-pcDNA3.1 (1.25 μg) or pcDNA3.1 (1.25 μg) (as a control) and hPSA2-Luc-pGL3 (plasmid consisting of the luciferase gene linked with the promoter of human prostate specific antigen; 1.25 μg) were transfected using LipofectamineTMLTX (6.25 μL) and PLUS reagent (2.5 μL) (Invitrogen, Carlsbad, CA, USA) into the cells according to the manufacturer’s protocol. At 6 h after the transfection, the cells were seeded on 96-well poly-L-lysine coated white-opaque plates at a density of 2.0 × 10^4^ cells/well. After overnight culture, the cells were treated with SARM-2f and dihydrotestosterone (DHT) (0.0001–1 nM) or dimethylsulfoxide (DMSO, as a solvent control) and cultured for an additional 24 h. Finally, the luciferase activities in the cells were measured using the Bright-Glo Luciferase assay kit (Promega, Madison, WI, USA) and a Wallac 1420 ARVO MX multi-label counter (Perkin Elmer, Waltham, MA, USA).

### Mammalian two-hybrid assays

We performed mammalian two-hybrid (M2H) assays (Promega Checkmate^™^) in 293T cells for androgen receptor-cofactor analysis as described previously [22]. In the present study, we evaluated cofactor recruitment to AR by M2H with 15 cofactors; prostate-derived Ets factor (PDEF), filamin A, alpha (FLNA), p21-activated kinase 6 (PAK6), TBP-associated factor 250 kD (TAFII250), protein inhibitor of activated STAT1 (PIAS1), protein inhibitor of activated STAT 3 (PIAS3), protein inhibitor of activated STAT y (PIASy), proline-rich nuclear receptor coactivator 2 (PNRC2), nuclear receptor subfamily 2, group C, member 2NR2C2 (TR4), steroid receptor coactivator 3 (SRC3), receptor of activated protein kinase C 1 (RACK1), steroid receptor coactivator 1 (SRC1), four and a half LIM domains 2 (FHL2), mothers against decapentaplegic homolog 3 (Smad3), and cytochrome co-oxidase subunit Vb (COX5B). DHT, testosterone, SARM-2f, and TSAA-291 (a steroidal SARM compound, prostetin) were added to 293T cells at 30, 100, and 300 nM. After 24 h culture, the luciferase activity was measured using the Bright-Glo Luciferase Assay System and a Wallac 1420 ARVO MX 1420 multi-label counter.

### Sexual behavior test

To confirm the sexual potency of male rats, 8-week-old male SD rats were kept together one-to-one in a cage for 4 days with 8-week-old Wistar Imamichi female rats that showed stable 4-day estrous cycles. All male rats were castrated under isoflurane anesthesia on the day after the mating. The male rats that had reproduction ability were selected for sexual behavior tests. Following nine weeks habituation after the castration, the rats were allocated to experimental groups according to body weight on day 0 (n = 10). SARM-2f was suspended in 0.5% MC solution and TP was dissolved in 20% BB corn oil. SARM-2f (0.03, 0.3, and 3 mg/kg SARM-2f po., QD, and BB oil sc. 3 times/week) or TP (0.03, 0.1, and 0.3 mg/kg of TP sc. 3 times/week, and 0.5% MC po., QD) were administrated to the castrated rats from day 1 for 3 weeks (n = 10). On the first day of dosing, DHEA pellets (1.5 mg/21-day release) were implanted to mimic the human body environment. The doses of SARM-2f and TP were in the range where the weight of the prostate and levator ani muscle linearly increased (Fig 1). On days 18–21, the rats in each group were housed overnight in a cage on a one-to-one basis with a female rat newly purchased that had exhibited stable estrous cycle and was in the proestrus phase on the day of mating. Female rats that continued to show diestrus over 10 days after the mating were judged to exhibit pseudo-pregnancy. The male sexual activity induced by the treatment was represented by the rate of female rats that became pseudopregnant. The rats were sacrificed under anesthesia after the sexual behavior test, and the levator ani muscle was collected for weighing.

### Mouse voluntary running activity

Physical activity was measured by determining daily running distance of wheel running. The running wheels (ENV-044 Mouse Low-Profile Wireless Running Wheel, Med Associates Inc., Fairfax, VT, USA; 15.5 cm circumference; 25° from horizontal plane) were located inside cages measuring 374 mm wide × 332 mm long × 147 mm high. Running activity was monitored through a wireless transmitter system using a Hub (DIG-804) located in the same animal colony room. The Hub was connected to a PC, and the number of rotations performed each hour was recorded during 2 weeks of treatment. Body composition was determined using EchoMRI-700 on the day before treatment. Mice were grouped by body weight, lean body mass, and fat mass into 5 groups (n = 8). Based on the plasma pharmacokinetics and the preliminary Hershberger assays (data not shown), the dose was set in the range where the weight of the levator ani muscle and the prostate increased presumably in a dose-dependent manner in mice. SARM-2f was resolved in DMSO: 1,3-butanediol (1:1) solution as 20 or 100 mg/mL, filled into an osmotic mini pump (28-day release, MODEL 1004, ALZET), and administered. The pumps filled with the vehicle were implanted into control sham-operated or castrated mice for 2 weeks (n = 8). The pump or testosterone pellets (1.5 mg /60-day release) were implanted into the mice under anesthesia. To mimic the human body environment, DHEA pellets (1.5 mg/60-day release) were also implanted into all castrated mice. The distance ran by each mouse was calculated as (3.14 × 15.5 cm × number of revolutions)/(100 cm per m) [23]. After a 2-week dosing period, the animals were sacrificed under anesthesia and their tissues were collected and weights measured.

### Mouse locomotive activity

SARM-2f and TP were administered to 10-week-old, male castrated C57BL/6J mice for 2 weeks (n = 12). Mice were grouped by body weight (n = 12). SARM-2f, TP, and DHEA were administered as for voluntary running. Locomotor activity of the mice was measured according to a previously described method with slight modification [24]. The locomotor activity in the home cage (185 mm wide × 332 mm long × 147 mm high) was assessed using the infrared sensor NS-AS01 (Neuroscience Co. Ltd., Tokyo, Japan), and counts of locomotor activity were collected in 10 min intervals for 48 h after the 2-week-treatments. Data were automatically analyzed using a computerized system and quantified by division into dark and light periods, which represent the active and resting periods for mice, respectively. Body weights were measured on day 0, 1, 8, 15, and 18. Food intake was measured using the food weight on day 1, 8, and 15. The mice were sacrificed under anesthesia after the measurement of locomotor activity, and the levator ani muscle was collected for weighing.

### Statistical analysis

In sexual behavior tests, Fisher's exact test was used for statistical analysis. In other experiments, comparisons between two groups were performed using the Student's t-test, and those between multiple groups were performed using the Williams or Dunnett's t-test. P-value < 0.05 was considered statistically significant.

## Results

### Tissue specificity of SARM-2f in the rat Hershberger assay

To confirm the tissue specificity of SARM-2f and to determine the appropriate dose for the sexual behavior test, a rat Hershberger assay was conducted. In this experiment, we subcutaneously implanted a 1.5 mg/21-day release pellet of DHEA in each rat to mimic the castrated condition in humans wherein DHEA exists as an adrenal androgen. SARM-2f increased the weights of the levator ani muscle and the prostate in a dose dependent manner (Fig 1A). The weight of the levator ani muscle reached the level of sham-operated non-castrated rats at the highest 3 doses (2, 10, or 50 mg/kg) (Fig 1A), whereas that of the prostate was 68% of the non-castrated level even at the highest dose of 50 mg/kg (Fig 1A). In comparison, TP showed a steeper rise in the dose response curve of both levator ani muscle and prostate weights (Fig 1B), and the difference between the levator ani muscle and the prostate curves was smaller than that of SARM-2f (Fig 1A and 1B).

### Tissue distribution of SARM-2f in rats

**Fig 2:**
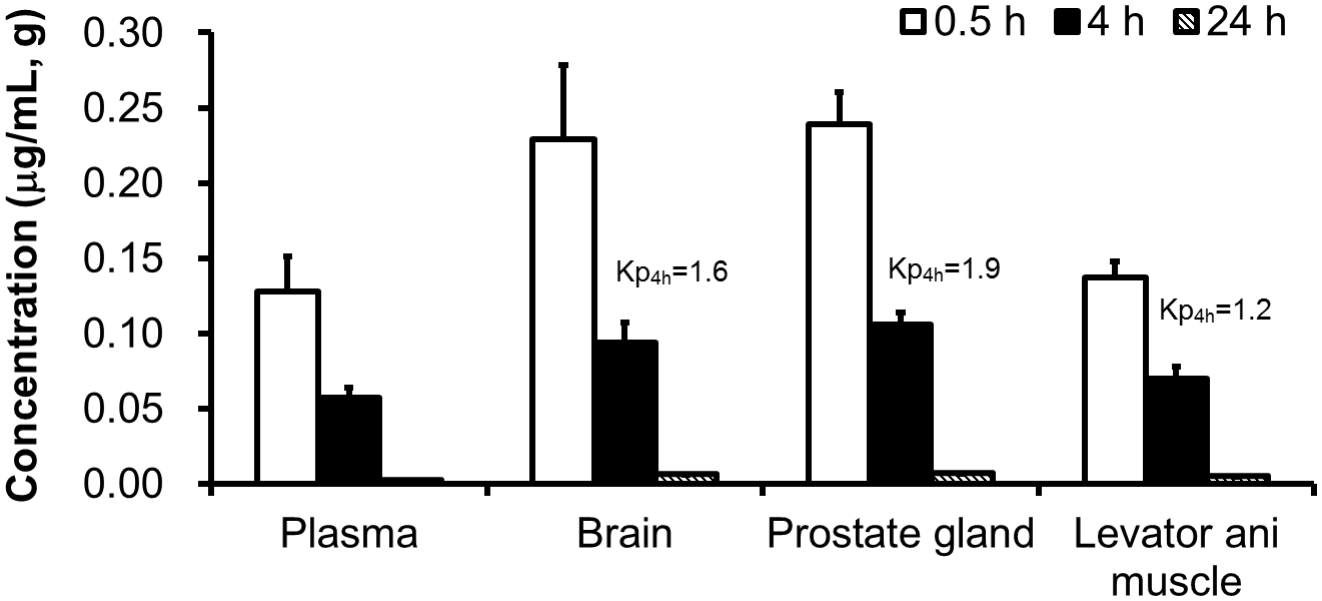
Concentrations of SARM-2f in tissues after oral administration at a dose of 0.75 mg/kg to rats. Data are shown as the means ± SD. (n = 3).

SARM-2f was not specifically distributed to the levator ani muscle; rather, it was more distributed to the prostate and the brain (Fig 2). The Kp was 1.9, 1.6, and 1.2 for the prostate, brain, and levator ani muscle, respectively, at 4 h after oral administration of SARM-2f to rats at the dose of 0.75 mg/kg (Fig 2). The same tendency was also observed at 0.5 and 24 h (Fig 2). Tissue-specific action of SARM was therefore not based on compound concentrations in tissues.

### Modulation of AR transcriptional activity by SARM-2f in human normal SkMC and PrEC

**Fig 3:**
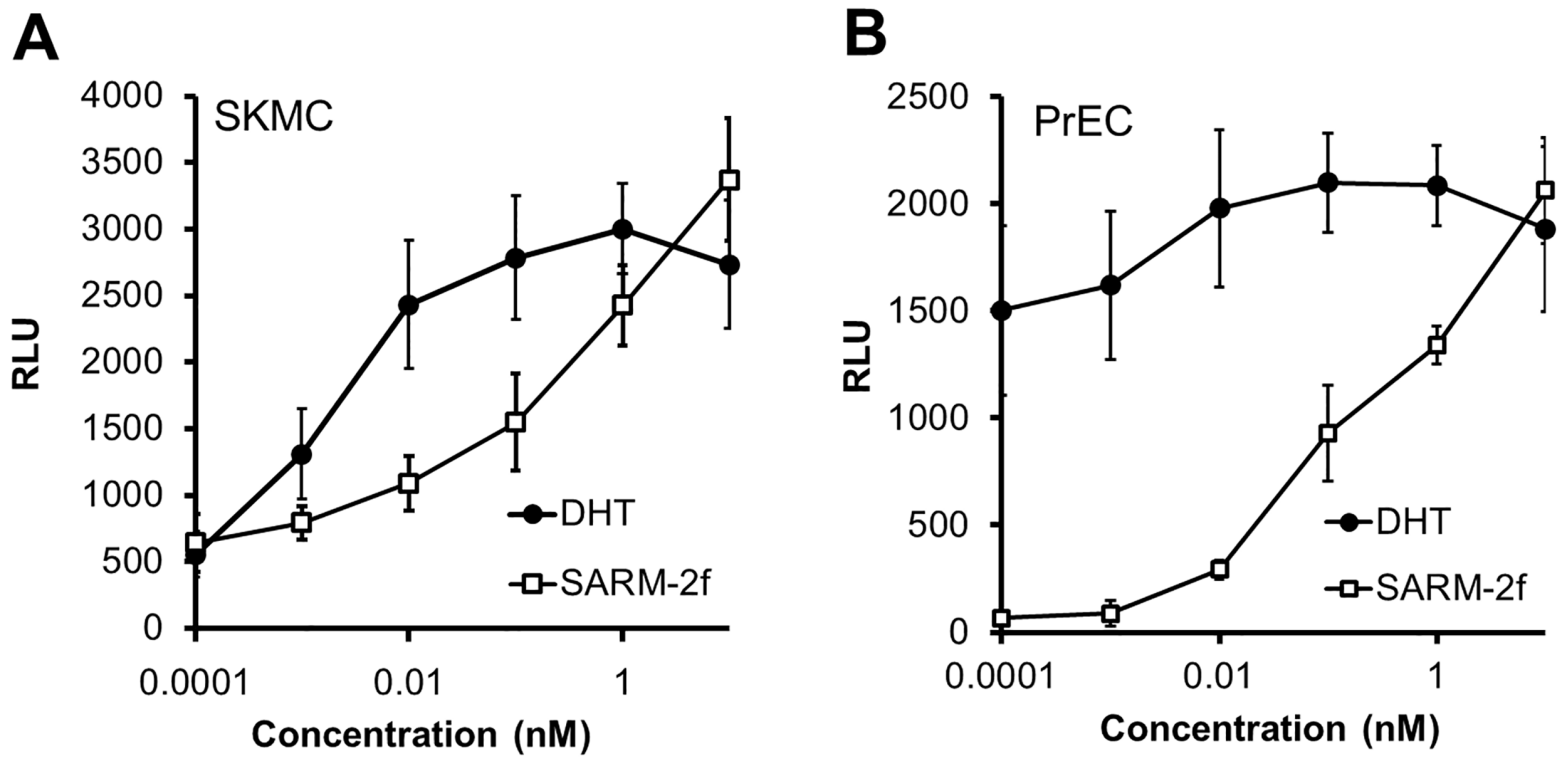
Cell context-dependent AR transcriptional activity of SARM-2f and DHT. The ability of compounds to influence AR-mediated transcriptional activation was examined in skeletal muscle cells (SkMC) (A) and prostate epithelial cells (PrEC) (B) by reporter assays as described in Material and methods. Data are shown as the means ± SD (n = 3).

To study the effect of SARM-2f on AR transcriptional activity in the prostate and muscle, we examined the activation of AR via the PSA promoter by SARM-2f and DHT in normal PrEC and normal SkMC. In SkMC, both SARM-2f and DHT stimulated the AR transcriptional activity in a concentration-dependent manner (Fig 3A). SARM-2f at the concentration of 0.001 nM showed AR stimulatory activity of 60.5% compared with DHT at 0.001 nM in SkMC (Fig 3A). In contrast, in PrEC, the AR activation by SARM-2f was markedly less than that by DHT at the concentrations of 0.0001 and 0.01 nM (Fig 3B). The AR stimulatory activity by SARM-2f at 0.001 nM was 5.4% compared with DHT at 0.001 nM in PrEC (Fig 3B).

### Different cofactor recruitment pattern between SARMs and AR full agonists

**Fig 4:**
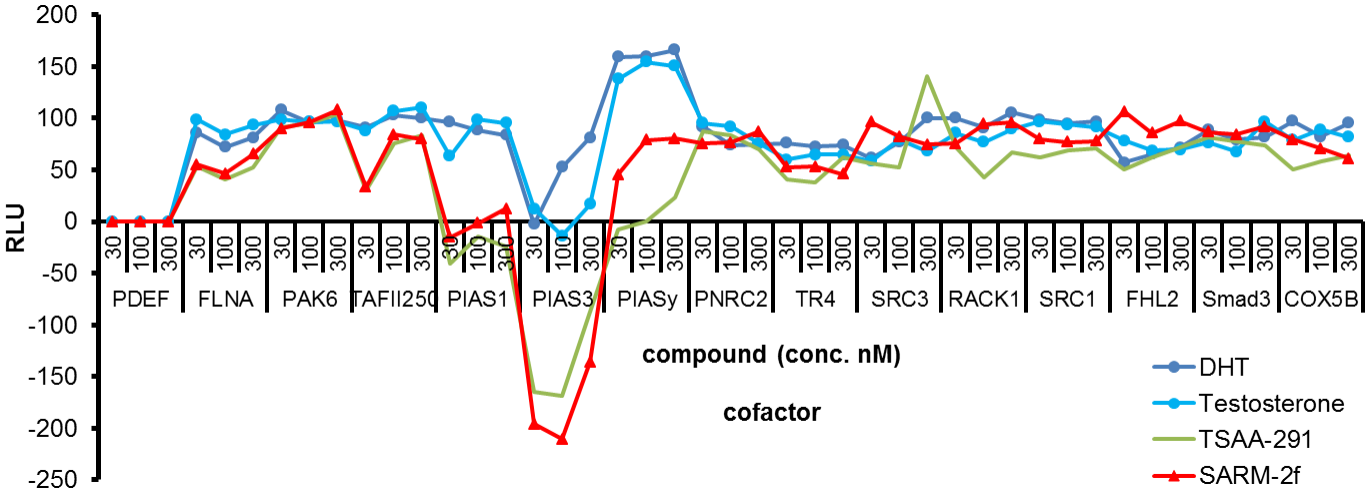
Cofactor recruitment analysis in 293T cells with DHT, testosterone, TSAA-291, and SARM-2f. Luciferase activity was measured after 24 h treatment with varying concentrations of DHT, testosterone, TSAA-291, and SARM-2f (30, 100, and 300 nM).

Testosterone and DHT are both potent AR full agonists and the cofactor recruitment pattern to AR when bound with these steroids was similar in the M2H system using 293T cells (Fig 4). SARM-2f and TSAA-291 are both SARMs, and the cofactor recruitment pattern between them was also similar (Fig 4). A difference between AR full agonists and SARMs was observed in PIAS family molecules including PIAS1, PIAS3, and PIASy (Fig 4).

### Induction of male sexual behavior by SARM-2f

**Fig 5:**
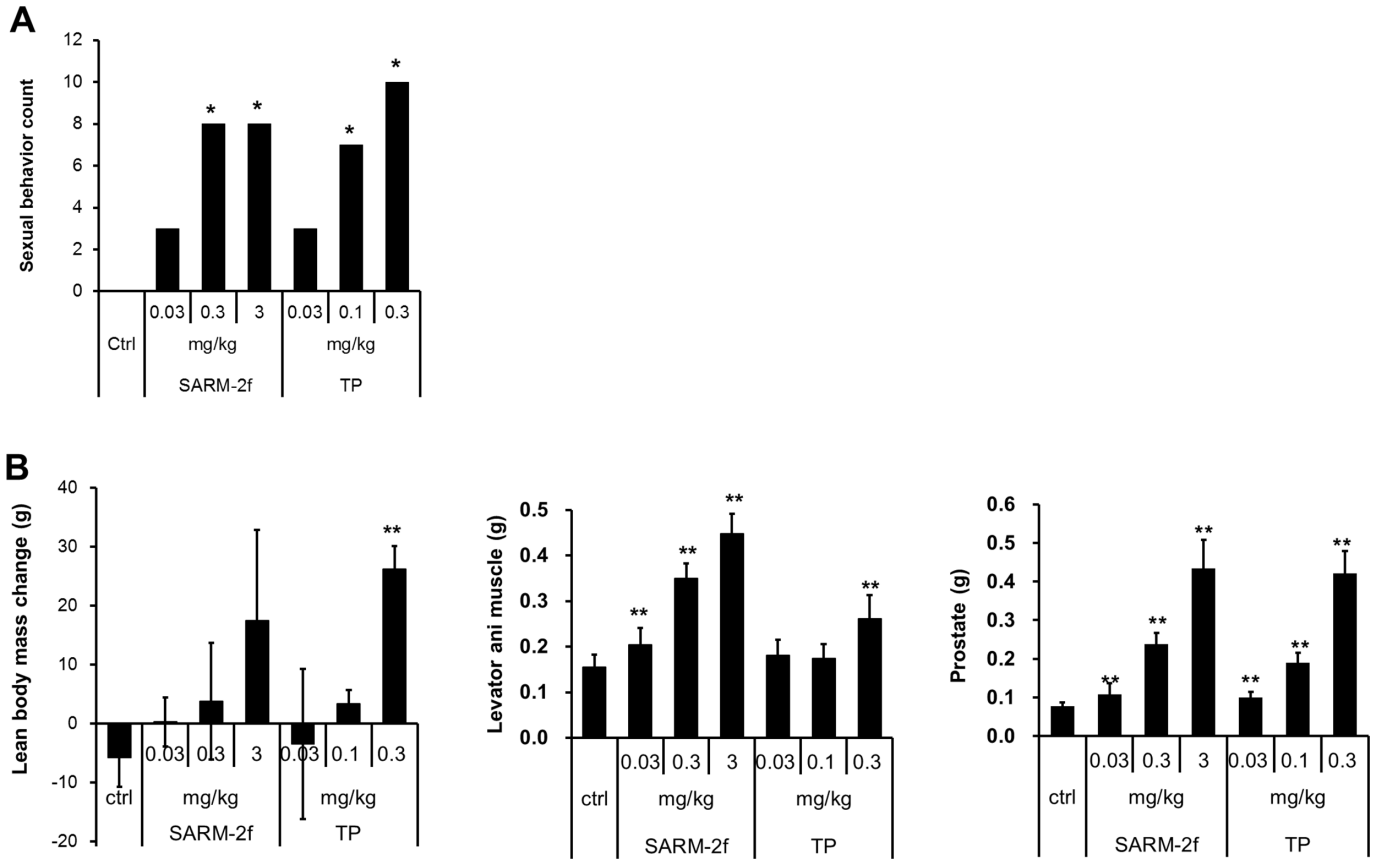
Induction of sexual behavior by SARM-2f and TP in castrated male rats. (A) Sexual behavior scores. (B) Lean body mass change and levator ani muscle weight (g), lean body mass. The drug was administered to castrated male rats for three weeks. Sexual behavior was determined by observation of the estrous cycle of female rats that had been mated with male rats treated with SARM-2f or TP at the end of the treatment period. (n = 10).*P < 0.05 vs. the control group with a Fishers' exact-test. **P < 0.005 vs. control group with the Williams test. Ctrl, Control vehicle group, TP, testosterone propionate.

None of the castrated rats treated with vehicle exhibited sexual activity (Fig 5A). TP induced sexual activity in a dose-dependent manner (0.03, 0.1, and 0.3 mg/kg) in castrated rats (Fig 5A) and all rats (10 of 10) treated with 0.3 mg/kg of TP induced sexual behavior (Fig 5A). SARM-2f also had a stimulating effect on sexual behavior in castrated male rats (Fig 5A). Sexual behavior was induced in 3 of 10 male rats by the dose of 0.03 mg/kg/day, and in 8 of 10 rats by 0.3 and 3 mg/kg/day of SARM-2f (Fig 5A). Levator ani muscle and prostate weight was increased by SARM-2f in a dose-dependent manner (Fig 5B).

### Induction of mouse voluntary running by SARM-2f

**Fig 6:**
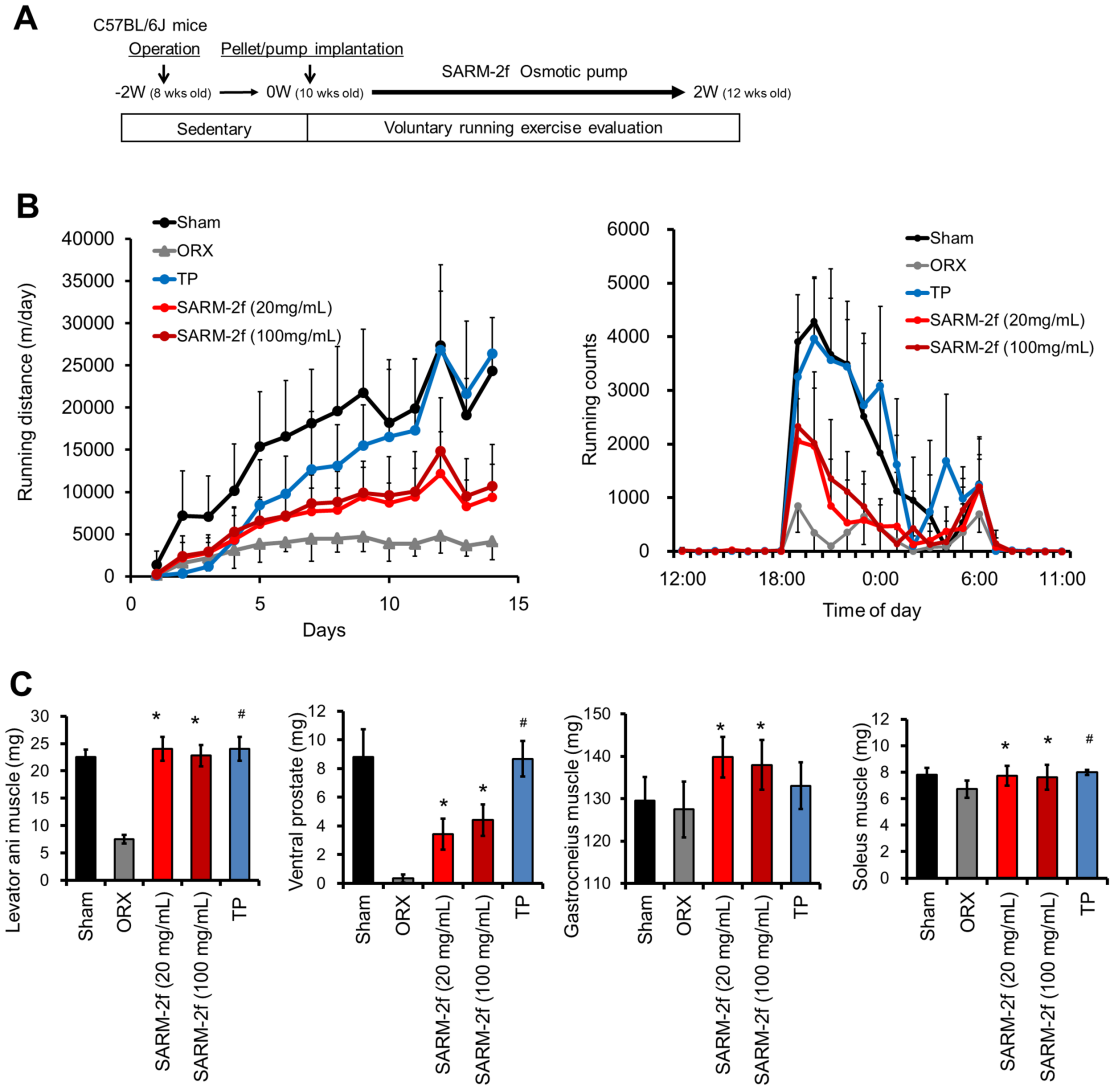
Effects of SARM-2f on mouse voluntary running. The outline of the voluntary running test is shown in Fig 6A. Running distance during 2 weeks of treatment with TP or SARM-2f was calculated by the formula described in the Materials and methods section (B). (B) Running count average in day13–14. (C) Levator ani muscle weight measured after 2-week dosing. The drug was administered to castrated mice with DHEA for 2 weeks. The measurement technique is described in the Materials and methods section. Data are shown as the means ± SD (n = 8). *P < 0.025 vs. the ORX (vehicle treated castrated mice) group using the Williams test. #P < 0.05 vs the ORX by student t-test.

In the first 3 days of the voluntary running, the running distance by each castrated mouse decreased progressively compared to that of sham-operated group mice irrespective of whether they received SARM-2f, TP, or vehicle (Fig 6B). Thereafter, the running distance per day by castrated control mice continued to be as short as 4500 m/day, whereas the running distance by castrated mice supplemented with TP (1.5 mg/60 days) increased progressively to 26,847 m/day on day 12, which was comparable to the running distance by the non-castrated sham mice (27,836 m/day) (Fig 6B). The result closely matched with the data previously reported in castrated mice with testosterone treatment in a voluntary running test [11]. SARM-2f (20, 100 mg/mL) -treated mice showed significantly increased running distance (12,177 and 14,870 m/day) on day 12, but not to the non-castrated mice level (Fig 6B). We analyzed the running profile of the mice in smaller time bins, as running counts (Fig 6B). All groups started to run at 19:00 (that is, at the start of the dark cycle), reached peak speeds by 20:00, and showed a 2^nd^ peak at 6:00, which was 1 h before lights on (Fig 6B). This movement pattern in the light and dark period was as reported previously [11]. The running counts were increased with the running distance as shown in Fig 6A, by TP and to a lesser extent by SARM-2f, compared with the castrated group (Fig 6B), although the bipolar pattern was not changed by SARM-2f or TP (Fig 6B). Both SARM-2f- and TP- treated mice exhibited 3-fold higher weights of the levator ani muscle compared to castrated mice, which reached the non-castrated group level (Fig 6C). The ventral prostate weight was increased 10- and 13-fold compared to the castrated group by 20 and 100 mg/mL SARM-2f, respectively, and 26-fold by TP (Fig 6C). The weight of the gastrocnemius muscle was increased 110% and 108% by SARM-2f compared to the castrated group by 20 and 100 mg/mL of SARM-2f, respectively, but not by TP (Fig 6C). The weight of the soleus muscle was increased 115% and 113% by SARM-2f compared to the castrated group by 20 and 100 mg/mL of SARM-2f, respectively, and 119% by TP (Fig 6C).

### Induction of mouse locomotor activity by SARM-2f

**Fig 7:**
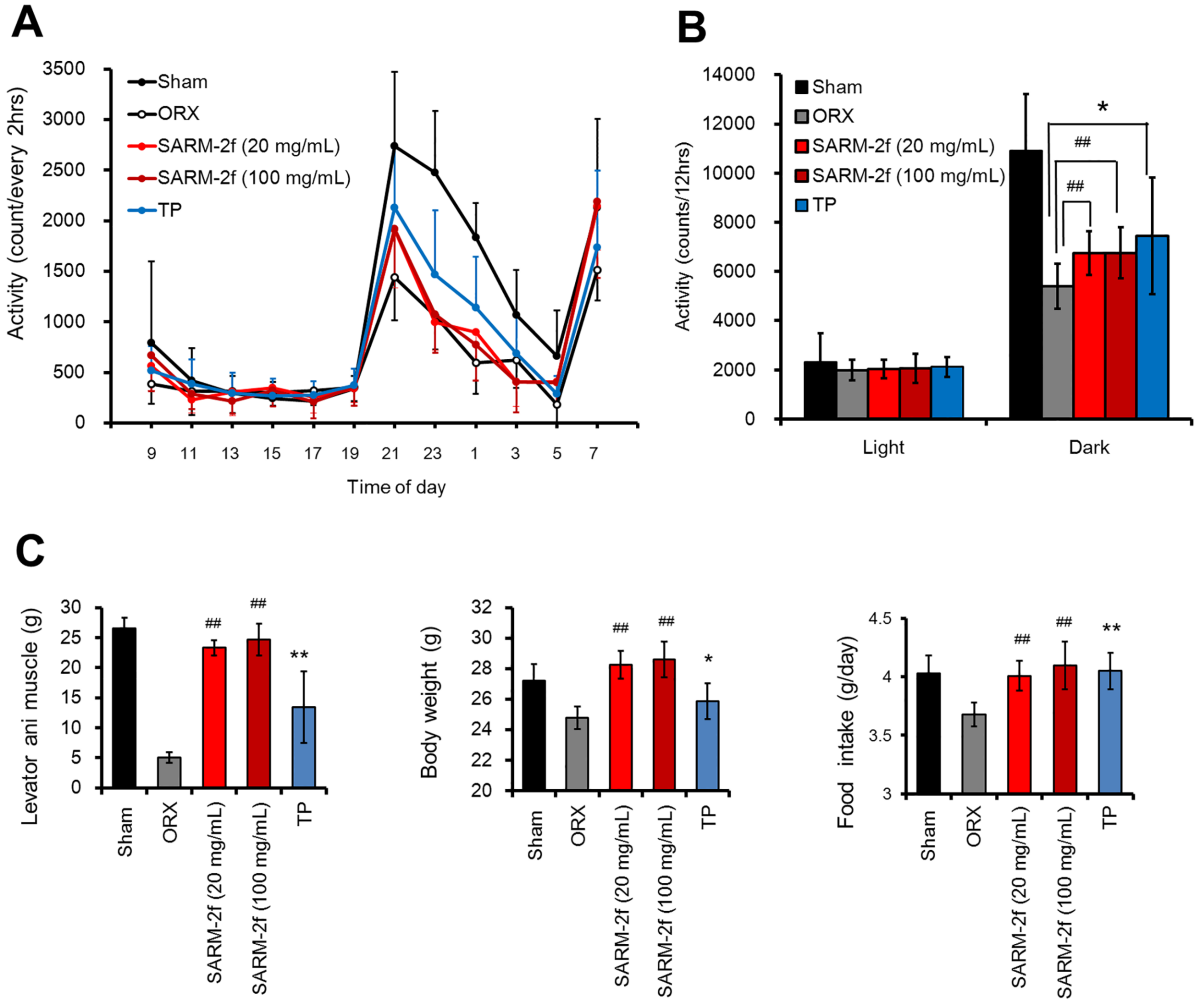
Locomotor activity. Distance travelled during 2 weeks of treatment with TP or SARM-2f was calculated by the formula described in the Materials and methods section (A). (B) Activity during the light or dark phase in day 13–14. (C) Levator ani muscle and body weight measured after 2-week dosing. Food intake was calculated during 2-week dosing. The drug was administered to castrated mice with DHEA for 2 weeks. The measurement technique has been described in the Methods section. Data are shown as the means ± SD (n = 8). ^##^P < 0.005 vs. ORX (vehicle treated castrated mice) group using a Williams test. *P < 0.05, **P < 0.01 vs. ORX group using a student t-test.

The distance travelled by castrated mice was found to be significantly reduced during the dark phase compared with that by non-castrated group mice (Fig 7A and 7B). SARM-2f (20 and 100 mg/mL) and TP (1.5 mg/60-day release) significantly enhanced spontaneous locomotive activities in the dark phase but not fully to the non-castrated level (Fig 7A and 7B). The levator ani muscle weight increased 4.6- and 4.9-fold compared to that of the castrated control group by SARM-2f at the doses of 20 and 100 mg/mL, respectively, and 2.6 times by TP (1.5 mg/60 days) (Fig 7C). Body weight and food intake were increased by SARM-2f (20 and 100 mg/mL) to the level of the non-castrated group (Fig 7C). TP increased food intake to the non-castrated level but not body weight (Fig 7C).

## Discussion

It was reported that SARM compounds increased the weight of skeletal muscles more than that of the prostate in rodent models [18, 25]. Our previous data also indicated that SARM-2f increased the weight of muscles more than that of the prostate in several rat and mouse models [19, 20]. However, the mechanisms of action for the tissue selectivity of SARM-2f have not been clarified, nor has whether the muscle weight increase by SARM-2f might lead to an improvement of physical activity yet been addressed.

In the present study, we examined the tissue distribution of SARM-2f in rats. However, the concentration in prostate was higher than that in the levator ani muscle, indicating that tissue distribution does not account for the tissue specific effect of SARM-2f. Next, we studied the modulation of AR transcriptional activity by SARM-2f in prostate and skeletal muscle cells. Consistent with the tissue specificity observed in *in vivo* study data [19, 20] (Fig 1), SARM-2f stimulated AR transcription to a larger extent in skeletal muscle cells than in prostate cells (Fig 3), suggesting that cell context-dependent modulation of AR transcription may account for the tissue specificity. M2H assays showed different cofactor recruitment patterns between SARMs and steroidal androgens, suggesting that conformational structure of AR when bound with SARM might be different from that with full AR agonists, which may lead to the cell context-dependent AR modulatory activity of SARMs. Notably, we confirmed that the cofactor recruitment pattern was quite similar between non-steroidal SARM-2f and steroidal TSAA-291, suggesting that the SARM specific cofactor-recruitment pattern arises from factors beyond structural difference.

We previously reported that the recruitment of some cofactors such as PIAS1 might be responsible for cell context-dependent AR transcriptional activity using another steroidal SARM compound, TSAA-291 [22]. PIAS1 is a coactivator of AR [22] and its expression level in prostate is higher than that in skeletal muscle [22]. The DHT-bound AR recruited PIAS whereas the SARM-2f-bound AR did not recruit PIAS in our M2H assays. These findings may explain, at least in part, the lower stimulus effect of SARM-2f on the prostate, compared with that of TP. Further studies are needed to fully explain the tissue selectivity.

Muscle wasting and decreased physical activity comprise unmet needs in clinical situations despite the fact that there are several candidate therapeutic agents under investigation for these diseases [26]. Testosterone has anabolic and functional effects both in *in vivo* rodent models and in clinical settings. Among other candidates, the SARM compound Ostaline increased muscle weights in phase III clinical studies in patients with non-small cell lung carcinoma cachexia [27, 28]. Nevertheless, the development of Ostaline for sarcopenia and cancer cachexia was discontinued because of the results of a step-ladder test, which is one of the functional recovery tests of muscle in patients with cancer cachexia [27]. The positive effect of Ostaline on muscle weight has been reported in preclinical studies, although its effect on physical function has not yet been reported [25]. In the present study, we showed that SARM-2f significantly induced sexual behavior, and improved voluntary running and locomotor activity in castrated rodents.

Conversely, SARM-2f did not improve mouse physical activity to the non-castrated levels in the three performance tests whereas TP both induced sexual behavior and increased running distance to the levels comparable to those of non-castrated male animals. Regarding sexual function, we previously reported that SARM-2f at doses of 0.5 and 5 mg/kg po. QD fully restored sexual activity in castrated male rats [19], indicating that SARM-2f has the potential of full recovery of sexual behavior. In the voluntary running test and the locomotor activity test, SARM-2f was less effective than TP. One possible reason for the difference of effect between TP and SARM-2f might be that testosterone is converted into estrogen. Circulating testosterone modulates anxiety levels and behavior [29] and estrogen is also necessary to increase locomotor activity in mice [30, 31]. Another reason might be the dose limitation of SARM-2f, as we could not increase the dose of SARM-2f because the concentration necessary for the osmotic pump system that was used in the two running tests was too high to avoid precipitation. If sufficient amount of SARM-2f exposure is possible, it may improve physical activity completely to the levels of non-castrated mice in voluntary running and locomotor activity. The other reason might be that the partial induction of running capability observed in this study represents the maximum effect of SARM-2f based on its pharmacological properties.

In our voluntary running test, the stimulus effect of SARM-2f on running distance was less potent than that of TP, whereas the effect of SARM-2f on skeletal muscle weight was more potent than TP, demonstrating no correlation between skeletal muscle weight and physical activity. This may be because voluntary running and locomotor activity are not only associated with muscular activity but also with motivation, an activity of the central nerve system, and because TP may stimulate motivation through estrogen or other metabolites.

It is reported that maximum muscle strength is proportional to muscle size [32]. However, there is also a study indicating that muscular strength does not reflect the muscle mass [33]. Although our study showed that muscle mass was increased by SARM-2f, we did not measure the muscle strength. Therefore, additional *ex vivo* studies are necessary to determine whether muscular strength is increased by SARM-2f.

Moreover, a relationship between the aerobic/oxidative capacity of the muscle and the running distance and activity has also been reported [34–36]. Although the present study showed improved running distance and locomotive activity by SARM-2f, we did not measure the aerobic/oxidative capacity of the muscle. This point should also be addressed in future studies.

Plasma testosterone level has been related to the muscle weight and cross section area (CSA) in a mouse model [37]. We did not measure CSA in this study, but CSA histological data would also be informative to eliminate the possibility of any adverse effects of SARM-2f treatment such as water retention and edema. It is assumed that some nuclear receptors are associated with water retention and edema. We reported previously that carcass weight was also increased by SARM-2f [20], and that there is selectivity against mineral corticoid receptor, which is a nuclear receptor related to water retention [19]. Based on these data, we consider that the increase in muscle weight by SARM-2f is unlikely to have stemmed from water retention in muscle.

In castrated humans, adrenal androgens exist in the plasma because these steroids are produced by the adrenal cortex. However, unlike human, rodent adrenal glands do not produce androgens. Therefore, we treated castrated rodents with low concentrations of androgens to mimic the hormonal environment in a castrated human body. In a previous study [19], the prostate weight was not increased by SARM-2f compared with that of the control group. In the present study, however, the prostate weight was increased by SARM-2f compared with that of the control group. The difference between the two studies is that TP (0.4 mg/kg sc qd) was added to all groups in the previous study [19], whereas DHEA (1.5 mg/21-day release pellet) was added in the present study. Other differences between the two studies include the castration duration and week-age of rats. The different baseline of prostate weight may reflect the presence or absence of prostate weight increase by SARM-2f. As SARM-2f did not increase the prostate weight to levels higher than those of gonadally intact rats in either condition, we believe that SARM-2f might not adversely affect the prostate.

In summary, we demonstrated that SARM-2f exerted cell context-dependent AR transcriptional activity, increased skeletal muscle weight with tissue selectivity, and improved sexual activity and locomotive activity in rodent models. Predicting the clinical outcome from preclinical data is still difficult and limiting. However, our data suggest that SARM-2f may constitute a novel therapeutic approach for patients with sarcopenia or cachexia with low testosterone levels.
